# Anisocoria after scopolamine transdermal patch contamination: A case report

**DOI:** 10.1097/MD.0000000000027887

**Published:** 2021-11-19

**Authors:** Jin-Ho Joo

**Affiliations:** Department of Ophthalmology, Kyung Hee University Hospital at Gangdong, Seoul, Korea.

**Keywords:** anisocoria, contamination, scopolamine transdermal patch

## Abstract

**Rationale:**

We report a case of anisocoria that occurred after contamination with a scopolamine transdermal patch, and introduce a diagnostic approach for anisocoria patients.

**Patient concerns:**

A 35-year-old woman with no past ophthalmologic history presented to the ophthalmology department complaining of a dilated pupil in the right eye. Corrected visual acuities was 20/20 in both eyes, and the intraocular pressures were 20 and 18 mm Hg in the right and left eye, respectively. The anterior chambers in both eyes were unremarkable on slit-lamp examination. The pupil size was 5.0 mm in the right eye and 2.0 mm in the left eye, and the extraocular muscles of both eyes were intact.

**Diagnosis:**

The patient neither did present with facial anhidrosis nor did she present with ptosis. Furthermore, as we did not observe dilatation lag in the smaller pupil, we applied 1% apraclonidine in the left eye in order to rule out Horner syndrome and did not observe dilatation of the pupil. We then applied 0.125% and 1% pilocarpine to exclude oculomotor nerve palsy; however, it could not be ruled out as constriction of pupil to 3.1 mm in the right eye was observed after applying 1% pilocarpine. Moreover, upon further investigation, we discovered that the patient had a scopolamine transdermal patch applied for 2 days prior to the clinic visit.

**Interventions:**

Artificial tears were administered and the patient was observed and monitored.

**Outcomes:**

The pupil size in the right eye gradually decreased to 4.5 mm on the second day of observation and to 3.6 mm on the fourth day of observation.

**Lessons:**

A detailed history of the use of medications such as scopolamine patches in patients with unilateral dilated pupils without vision loss is of utmost importance. We report the exclusion of important diseases using pilocarpine and apraclonidine hydrochloride. It was confirmed that improvement naturally occurs over time.

## 1 Introduction

A substance called scopolamine or hyoscine is a parasympathetic inhibitor that competitively inhibits the muscarinic acetylcholine receptor. The chemical structure of scopolamine is known to be very similar to that of atropine, and when instilled into the eye, it induces a mydriasis and causes sedation, antiemesis, and memory impairment^[1,2]^ In particular, scopolamine is the first commercialized transdermal absorption treatment; is applied to the skin behind the ear, which is relatively easily absorbed; and is marketed for the prevention of motion sickness and nausea and vomiting after surgery.^[2,3]^

Common side effects of the drug include dry mouth, dry skin, drowsiness, dilated pupils, and, in serious cases, urinary dysfunction, delirium, and glaucoma.^[4]^ This report aims to elucidate a case of a patient with dilated pupils without loss of vision and with dilated pupils due to scopolamine patches (scopolamine 1.5 mg, Kimite Patch; Myungmoon Pharm Co., Seoul, Korea) after differential examination and history taking.

Informed written consent was obtained from the patient for publication of this case report and accompanying images. No ethical approval was obtained because this study is retrospective case report and did not involve a prospective evaluation.

## 2 Case report

A 35-year-old woman with an unremarkable ophthalmologic history presented to the ophthalmology department complaining of a dilated pupil in the right eye while washing her face. The patient had no pertinent medication use history except for diuretics and steroid injection into the eardrum because of low-pitched hearing loss during a consult at the otolaryngology department.

**Figure 1:**
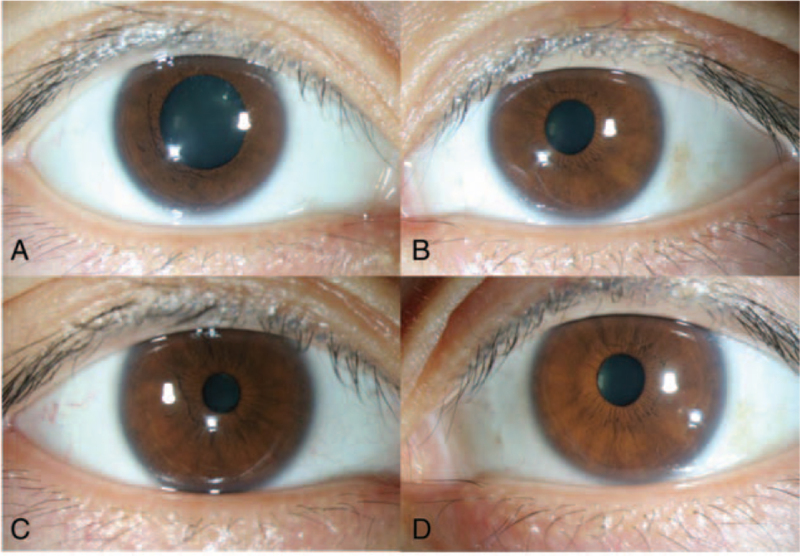
Anterior photographs of the dilated pupil in the right eye at the initial examination and a reduction in the pupil size after application of 1% pilocarpine. At initial presentation, the diameter of the pupil is 5 mm in the right eye (A) and 2 mm in the left eye (B). After applying 1% pilocarpine in the right eye, the size of the pupil is reduced (C) in comparison to the pupil size of the left eye (D).

On examination, the best-corrected distance visual acuity was 20/20 in both eyes. Her intraocular pressures measured using a noncontact tonometer were 20 and 18 mm Hg in the right and left eyes, respectively. Automated refraction revealed a sphere of −1.50, cylinder of −0.25, axis of 171 in the right eye and sphere −1.00, cylinder −0.75, axis of 162 in the left eye. Slit lamp microscopy revealed no specific findings in the cornea and conjunctiva, and anterior chamber inflammatory cells were not observed in either eye, and there was no media opacity such as cataracts. No specific findings were observed on the color fundus examination. The anterior depth of both eyes was the same when measured visually, but the difference was found to be 5.0 mm in the right eye and 2.0 mm in the left eye (Fig. 1A, B).

The pupillary reflex in the left eye was normal, but the right eye was sluggish, and there was no pupil dilatation delay in the left eye in the dark, and relative afferent pupillary movement disorder was not observed in either eye. To determine the cause of the dilated pupil, pilocarpine (Isopto Carpine 2%, Novartis, Basel, Switzerland) diluted in 0.125% was administered to the right eye; however, there was no miosis. Next, when 1% pilocarpine was administered, it was confirmed that the pupil of the right eye was reduced to a size similar to that of the left eye (Fig. 1C, D). We suspected an oculomotor nerve (third cranial nerve) palsy, but since ptosis and ocular movement disorders in both eyes were not confirmed, it could be excluded to some extent. In the left eye with a small pupil diameter, 0.5% apraclonidine hydrochloride (Iopidine Eye Drops 1%, Novartis, Basel, Switzerland), an alpha (α)-2 agonist, was administered to confirm that no change in the pupil occurred and Horner syndrome was excluded. The patient did not complain of anhidrosis, but a sympathetic skin response test, Valsalva maneuver, heart rate variation, and heart rate difference to deep breathing were performed to check the abnormal state of the autonomic nervous system (ANS); however, no specific concerns were observed.

After the examination, the specific cause of anisocoria could not be identified, and the history of drug use mentioned by the patient was later confirmed. She was seen by a gastroenterologist with symptoms of nausea and vomiting 1 month before her visit. However, the nausea symptoms recurred, and she then attached a scopolamine patch (Kimite Patch; Myungmoon Pharm Co., Seoul, Korea) 2 days before her visit, and she washed her face in that state. After washing her face on the morning of the day of her visit to our department, she noted that the pupil of her right eye was dilated; therefore, we suspected that the scopolamine patch had contaminated the eye during rubbing or washing. Accordingly, artificial tears were administered and follow-up care was performed. Furthermore, it was difficult to completely exclude other diagnosis other than drugs for the anisocoria, especially oculomotor nerve palsy; therefore, brain magnetic resonance imaging (MRI) and magnetic resonance angiography (MRA) were performed. However, no specific abnormal findings were observed.

**Figure 2:**
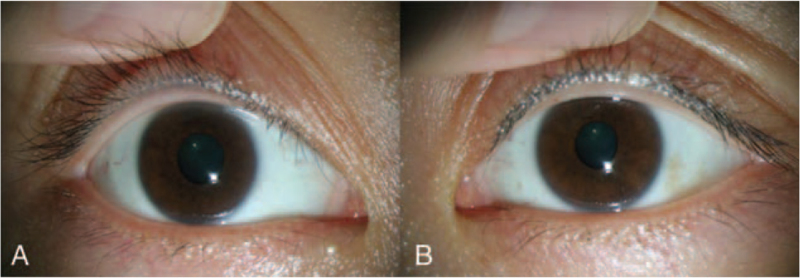
Anterior photographs of the right eye pupil (A) showing a return to its normal size when compared to the pupil in the left eye (B) after 2 days.

The pupil diameter measured on the first day of follow-up was 4.5 mm in the right eye, which was decreased by 0.5 mm compared to that on the previous day. Light reflection of the right eye was still sluggish, but proximity reflection was confirmed. The pupil diameters measured on the second day were 3.1 mm in the right eye and 2.6 mm in the left, confirming that the pupil diameter in the right eye had decreased (Fig. 2A, B). The patient was satisfied with the condition improved without any special treatment.

## 3 Discussion

Anisocoria is a common condition, defined by a difference of 0.4 mm or more between the sizes of the pupils of both eyes.^[5]^ Although the size of the pupils varies from person to person, if the pupil sizes of both eyes in a person are significantly different from each other, a detailed examination of the pupils is necessary. First, when anisocoria occurs, it is necessary to determine whether mydriasis or miosis has occurred.

Horner syndrome may be suspected if there is facial anemia or ptosis, 1 pupil is constricted, and there is dilatation delay in the dilated pupil in a dark environment.^[6]^ This can be evaluated by administering 0.5% to 1% apraclonidine hydrochloride or 4% to 10% cocaine to both eyes. When the drooping eyelids rise and the pupil dilates because of the administration of 0.5% apraclonidine hydrochloride or 10% cocaine, Horner syndrome should be strongly suspected. Instillation of 1% hydroxyamphetamine is performed to determine whether it is a preneuronal lesion or a postneuronal lesion. If mydriasis occurs, central or preganglionic Horner syndrome should be suspected; otherwise, postganglionic Horner syndrome should be considered. Brain MRI and chest computed tomography or MRI should be performed expeditiously to identify the cause of the disease.^[7]^

**Figure 3:**
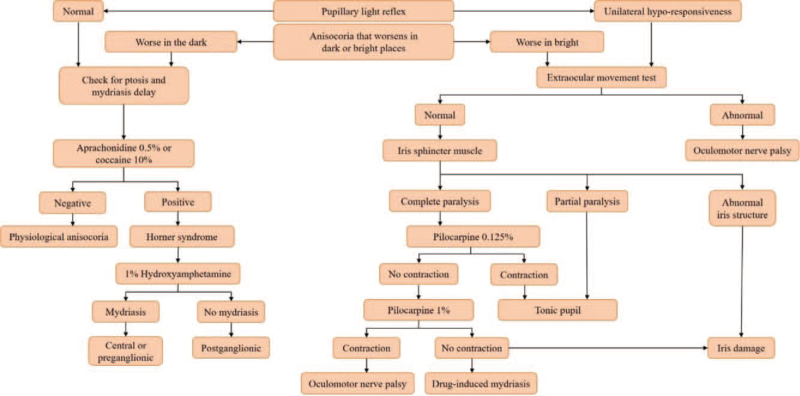
Diagnostic approach of anisocoria.

In contrast to Horner syndrome, when monocular mydriasis occurs, a tonic pupil can be suspected. Diagnosis is possible when the dilation is caused by hypersensitivity to pilocarpine diluted in 0.05% to 0.15%. In addition, if sector palsy of the sphincter is observed, it could help in the diagnosis. However, if the reduction does not occur even after instillation, pilocarpine is undiluted to 1% to 2% a second time, and if reduction occurs, oculomotor nerve palsy is considered. However, if reduction still does not occur, a drug reaction is considered.^[8]^ In the case of oculomotor nerve palsy, even if the pilocarpine test is positive, ptosis or eye movement disorder is mainly found together; if these symptoms occur together, brain MRI and MRA should be performed.^[9]^ The diagnostic approach of anisocoria is shown in Figure 3.

In the case of anisocoria due to a drug reaction, the drug directly contacts the eye or is absorbed into the human body in other ways, and the drug affects the central nervous system or ANS. Drugs that have been reported in the academe include scopolamine transdermal patches, bronchodilator nebulizers such as ipratropium, and antiperspirant agents containing glycopyrrolate, which cause mydriasis ^[4,10,11]^. Pilocarpine, opium, and organophosphate insecticides are known to cause miosis.^[12]^

The pupil of the body is regulated by the ANS. When the alpha-1 adrenergic receptor (α-1 receptor) is stimulated by the sympathetic nerve, the iris dilator muscle contracts and the pupil enlarges; conversely, when the muscarinic receptor (M3 receptor) is stimulated by the parasympathetic nerve, the iris sphincter muscle and the ciliary muscles contract, causing the iris to contract.^[9]^ In particular, when the scopolamine transdermal patch is used and then absorbed by the eyes through contaminated hands, the scopolamine component inhibits the muscarinic receptors and prevents the iris from moving, causing pupil dilation.^[1]^

Although the side effects of transdermal scopolamine patches are less compared to when they are systemically administered, drowsiness and dry mouth may occur,^[13]^ and anisocoria and cycloplegia are considered to be important issues in the ophthalmic field. The mydriatic effect of 0.5% scopolamine was reached 20 to 30 minutes after instillation, and the duration was 3 to 7 days.^[13]^ Anisocoria is not seen in both eyes; therefore, it is reasonable to assume that hand contact is the cause.

The patient in this case report visited the hospital with complaints of anisocoria, and to first rule out serious diseases such as Horner syndrome and oculomotor nerve palsy, basic history taking and ophthalmologic examination using drugs such as pilocarpine and apraclonidine hydrochloride were performed. The results are depicted in the photographs. Specific symptoms such as ptosis and oculomotor disorders were not identified, but the last administration of undiluted pilocarpine caused the pupil to migrate; therefore, oculomotor nerve palsy could not be completely excluded. For an accurate diagnosis, additional studies such as MRI and MRA were performed, and no unusual features were observed. Then, the pupil size of the dilated right eye gradually decreased, revealing that it was a drug reaction. In this case, when undiluted pilocarpine was used, the pupil was partially constricted, and the oculomoter nerve palsy could not be completely excluded, making it difficult to diagnose. Since the scopolameina transdermal patch was applied 2 days ago, it is thought that it was because the mydriatic effect decreased as the concentration of the drug decreased. Therefore, the importance of history taking is highlighted.

Anisocoria caused by transdermal scopolamine patches can cause considerable confusion for the physician and, in some cases, lead to unnecessary neurological examinations. We report the exclusion of important diseases using pilocarpine and apraclonidine hydrochloride with detailed photographs of the occurrence of anisocoria. It is important to exclude serious diseases by performing tests such as MRI and angiography; however, excluding drug reactions through a detailed history of drug use that can affect the pupils is also crucial.

## Acknowledgments

We would like to thank Editage (www.editage.co.kr) for the English language editing.

## Author contributions

**Conceptualization:** Jin-Ho Joo.

**Data curation:** Jin-Ho Joo.

**Formal analysis:** Jin-Ho Joo.

**Funding acquisition:** Jin-Ho Joo.

**Investigation:** Jin-Ho Joo.

**Methodology:** Jin-Ho Joo.

**Project administration:** Jin-Ho Joo.

**Supervision:** Jin-Ho Joo.

**Writing – original draft:** Jin-Ho Joo.

**Writing – review & editing:** Jin-Ho Joo.
